# Amperometric Non-Enzymatic Hydrogen Peroxide Sensor Based on Aligned Zinc Oxide Nanorods

**DOI:** 10.3390/s16071004

**Published:** 2016-06-29

**Authors:** Naif H. Al-Hardan, Muhammad Azmi Abdul Hamid, Roslinda Shamsudin, Norinsan Kamil Othman, Lim Kar Keng

**Affiliations:** School of Applied Physics, Faculty of Science & Technology, Universiti Kebangsaan Malaysia (UKM), Bangi 43600, Malaysia; linda@ukm.edu.my (R.S.); insan@ukm.edu.my (N.K.O.); karkeng.iamkklim@gmail.com (L.K.K.)

**Keywords:** non-enzymatic biosensor, zinc oxide, nanorods, hydrogen peroxide

## Abstract

Zinc oxide (ZnO) nanorods (NRs) have been synthesized via the hydrothermal process. The NRs were grown over a conductive glass substrate. A non-enzymatic electrochemical sensor for hydrogen peroxide (H_2_O_2_), based on the prepared ZnO NRs, was examined through the use of current-voltage measurements. The measured currents, as a function of H_2_O_2_ concentrations ranging from 10 μM to 700 μM, revealed two distinct behaviours and good performance, with a lower detection limit (LOD) of 42 μM for the low range of H_2_O_2_ concentrations (first region), and a LOD of 143.5 μM for the higher range of H_2_O_2_ concentrations (second region). The prepared ZnO NRs show excellent electrocatalytic activity. This enables a measurable and stable output current. The results were correlated with the oxidation process of the H_2_O_2_ and revealed a good performance for the ZnO NR non-enzymatic H_2_O_2_ sensor.

## 1. Introduction

Hydrogen peroxide (H_2_O_2_) plays an important role in environmental monitoring, clinical diagnosis, and in the food industry [1]. The monitoring of H_2_O_2_ is also important because H_2_O_2_ results from many biological targets, such as glucose and lactose [2]. It has been noted that diseases such as Alzheimer’s, Parkinson’s, and other central nervous system diseases are caused by excessive amounts of H_2_O_2_ [1]. It has been further noted that H_2_O_2_ contributes to the formation of acid rain, which makes the determination of H_2_O_2_ levels a crucial task in the fields of physiology, pathology, and the environment [3,4].

Several methods have been used and developed to detect H_2_O_2_, such as titrimetry, spectrophotometry, chemiluminescence, and electrochemical methods [5,6]. However, these methods have certain drawbacks, such as expensive reagents, long analysis times, and poor long-term stability [7]. Electrochemical approaches to the measurement of H_2_O_2_ have gained increasing attention due to their high sensitivity, low cost, low power consumption, and capability of practical applications. Metal-oxide semiconductors are among those materials with promising behaviour in the field of electrochemical biosensor applications. Metal-oxide semiconductors such as tin oxide [2], tungsten oxide [5], copper oxide [6], titanium dioxide [8,9], magnesium oxide [10], cuprous oxide [11] and zinc oxide (ZnO) have demonstrated outstanding performance in the biosensor and chemical sensor fields [7,12]. High catalytic efficiency, biocompatibility, and chemical stability in physiological environments, as well as nontoxicity, make ZnO a promising candidate for such applications [13,14]. Thus far, ZnO nanostructures have been employed as biosensors to detect several analytes, such as uric acid [15], glucose [16], and phenolic compounds [17].

In addition, ZnO has been used as an enzymatic-based biosensor for redox enzymes such as cortisol [13], cytochrome c [14], cholesterol oxidase [18] and horseradish peroxidase [19]. Furthermore, ZnO enzymatic-based biosensors have been extensively employed for H_2_O_2_ detection. Results tend to show high sensitivity and excellent selectivity for different analytes [20], although they are still the subject of intensive study. Enzyme immobilisation and its stability on an electrode surface is a crucial factor, as the enzyme can be easily damaged during the fabrication process and testing [6,21]. Moreover, enzymatic-based biosensors are only able to work effectively under proper conditions and are thus not conducive to a wide range of applications. Furthermore, enzymatic-based biosensors generally use expensive materials, such as multi-walled carbon nanotubes (MWCNTs) [22], gold, silver, palladium, and platinum [23], and enzyme immobilisation involves multiple complex steps [24]. Non-enzymatic biosensors, on the other hand, have been suggested for sensing several analyte species [25,26]. Many published works have focused on enzymatic biosensors based on ZnO, while relatively fewer works have been concerned with non-enzymatic ZnO biosensors [27,28,29].

The simple growth of ZnO nanostructures over a wide range of substrates with high crystallinity make it favourable over many other compounds. Both physical and chemical deposition techniques were used to synthesise ZnO nanostructures. Techniques include sol-gel, thermal evaporation [30], electrochemical deposition [31], and hydrothermal methods. The latter show high repeatability and are considered a low temperature process [12].

In this paper, we have fabricated an enzyme-free H_2_O_2_ electrochemical biosensor based on ZnO nanorods (NRs) grown on a conductive glass substrate via the hydrothermal method. The produced ZnO NRs can be directly used as sensor electrodes without any additional processing, such as surface modification or immobilisation of expensive enzymes. Moreover, with a NR structure, a large surface-to-volume ratio can be achieved, which will improve the efficiency of electron transport. The produced ZnO NRs will initially be characterised using an X-ray diffractometer for the phase structure. A ZnO NR/aluminium-doped ZnO (AZO) electrode was tested in a two-electrode method in the presence of H_2_O_2_. The concentration of the analyte ranged from 10 μM to 700 μM.

## 2. Materials and Methods

The ZnO NRs were grown on a commercial glass slide coated with a conductive layer of aluminium-doped ZnO (AZO) with a sheet resistance of 10 Ω/square. Initially, the substrates were cleaned by soaking them in beakers filled with acetone, isopropanol, and deionized (DI) water for 10 min in an ultrasonic bath. The growth process was explained in detail in our previous work [12]. Here, we will summarize the process. Equimolar amounts of 25 mM aqueous solutions of zinc nitrate hexahydrate (Zn(NO_3_)_2_·6H_2_O) and hexamethylenetetramine (C_6_H_12_N_4_) were dissolved in 200 mL of DI water and stirred continuously for 1 h. Substrates were vertically immersed in the solution in a screw-capped bottle, and then the bottle was placed into a laboratory oven at a temperature of 95 °C for 4 h. During this process, the Zn^2+^ species reacted with OH^−^ to form the Zn(OH)_2_ intermediate complex, which decomposed to ZnO at high temperatures. Prepared samples were then washed several times with DI water before being dried at 100 °C and then post-annealed for 1 h at 400 °C under ambient atmospheric conditions.

The sensing characteristics of the prepared AZO/ZnO NRs electrode toward H_2_O_2_ were examined using a 6517A electrometer (Keithley Instruments, Inc., Cleveland, OH, USA). A two-electrode cell was used for measuring the I-V characteristics, with the AZO/ZnO acting as the sensing electrode (working electrode) with an active area of 0.5 cm^2^, and a platinum wire (diameter of 0.5 mm) being used as the counter-electrode. The distance between the electrodes was fixed at 1 cm. The current response was measured by applying a voltage in the range of 0.1 V to 6 V. Measurements were commenced after 20 s with the addition of H_2_O_2_ (35.0% from Sigma-Aldrich (M) Sdn. Bhd. Kuala Lumpur, Malaysia) to the DI water (resistivity = 18 MΩ-cm). The solutions were prepared by mixing a known amount of H_2_O_2_ with DI water in different concentrations ranging from 10 μM to 700 μM. A magnetic bar with a stirrer was used to achieve homogeneity of the solution in a short time. Measurements were performed under ambient atmospheric conditions (23 °C and 50% relative humidity).

The prepared ZnO NR samples were characterised for phase structures via an X-ray diffractometer (D8 ADVANCE, Bruker, Billerica, MA, USA), which was operated at a voltage of 60 kV with a copper target. The scanning Bragg angle was recorded in the range of 20° to 60°).

## 3. Results and Discussion

### 3.1. Structure and Morphology of the ZnO NRs

**Figure 1:**
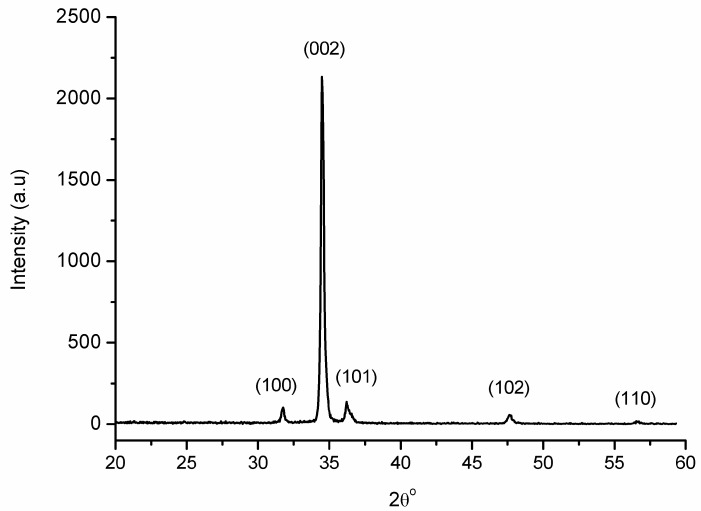
The XRD pattern of the prepared ZnO NRs on the AZO substrate.

The pattern of X-ray diffraction (XRD) for the prepared ZnO layer is depicted in Figure 1. The pattern showed a predominant peak at a Bragg angle equal to 34.48°, which corresponds to the (002) phase of the ZnO hexagonal structure. The sharp diffraction peak from the (002) plane indicated the excellent crystallinity of the prepared sample. Several low intensity peaks were also noticed in the pattern, which can be well indexed to the hexagonal ZnO structure according to the database JCPDS No. 36-1451. No diffraction peaks of impurities were observed, indicating the high purity of the final products. The surface morphology of the hydrothermally-grown ZnO was similar to our previous results and can be seen in our published article [12].

### 3.2. The Sensing Performance of ZnO NR

#### 3.2.1. The I-V Response to Hydrogen Peroxide

**Figure 2:**
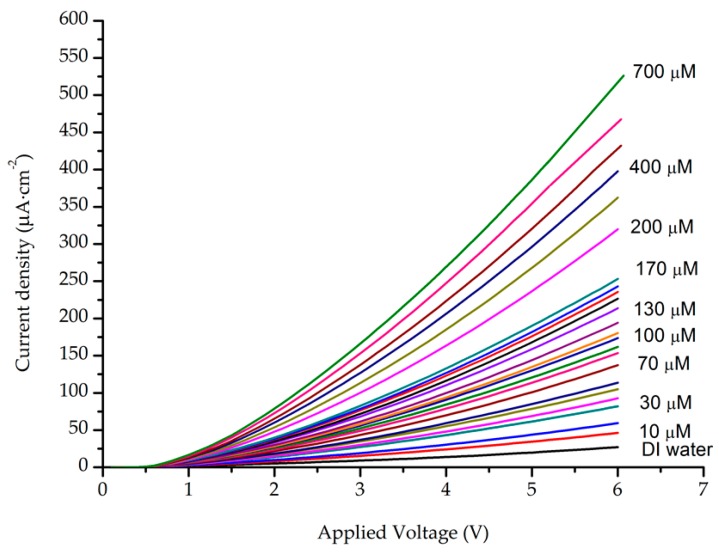
I-V characteristics of the ZnO NR sensing electrode for H_2_O_2_ in concentrations ranging from 10 μM to 700 μM.

The sensing characteristics of AZO/ZnO electrodes toward H_2_O_2_ concentrations were tested at room temperature. The I-V characteristics of the prepared sensing electrode are shown in Figure 2. As can be seen in the figure, a low current density was detected in pure DI water, but the current density was continuously increased with the addition of H_2_O_2_ to the DI water. This increase in the current density is due to the generation of ions with the addition of the H_2_O_2_. This can be understood in terms of the following reactions [32]:

(1) $$\begin{array}{l} O_{2}(\text{dissolved})\;\to \;O_{2}(\text{adsorbed})\\ O_{2}(\text{adsorbed})+e^{-}\;\to \;O_{2}^{-}(\text{adsorbed})\\ 2H_{2}O_{2}+O_{2}^{-}\;\to \;2H_{2}O+2O_{2}+2e^{-} \end{array}$$

According to the above reactions, the dissolved oxygen molecules are chemisorbed into sites covering the surface of the ZnO NRs. The electrons from the conduction band are attracted to the oxygen-adsorbed molecules, which results in a reduction in the charge carrier’s density. Consequently, a barrier height near the surface is formed and the resistivity of the ZnO NRs is increased [32]. The addition of the H_2_O_2_ to the DI water results in the release of negative ions (e^−^). More H_2_O_2_ added to the solution will result in the release of more electrons, thereby decreasing the barrier height. Subsequently, the resistivity will decrease, and the measured current density will increase.

**Figure 3:**
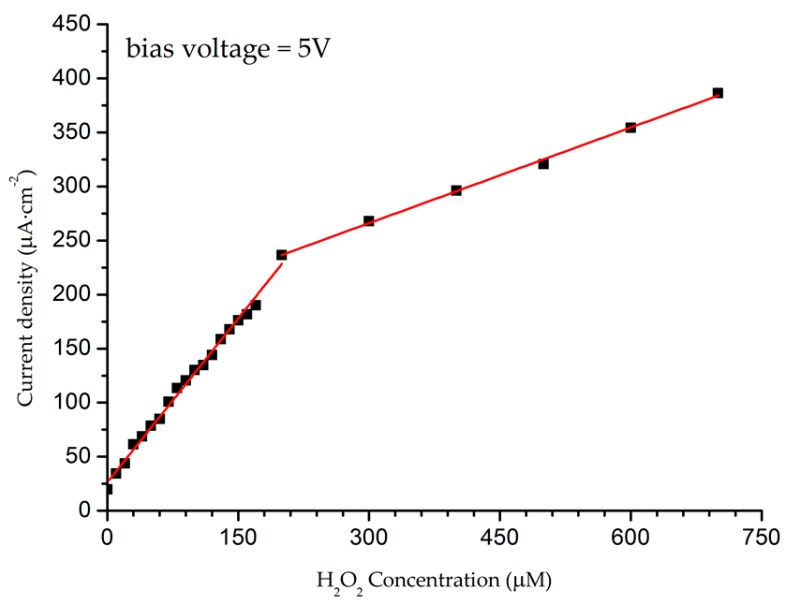
Response of the AZO/ZnO NR sensing electrode at bias voltages of 5 V in a diluted solution of H_2_O_2_ in concentrations ranging from 10 μM to 700 μM.

**Figure 4:**
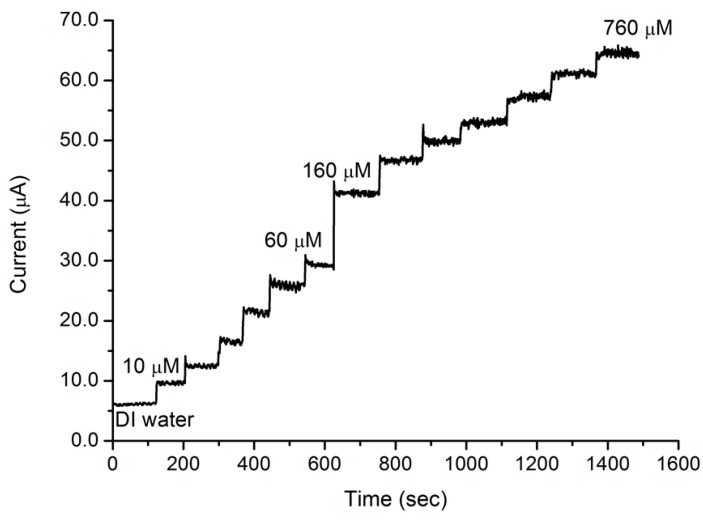
The amperometric response of the ZnO NR/AZO electrode upon successive additions of H_2_O_2_. The bias voltage was fixed at 5 V.

We chose data of the measured current density in relation to H_2_O_2_ concentrations at a fixed bias voltage of 5 V to evaluate the sensitivity of the prepared sensing electrode (see Figure 2). Figure 3 depicts the results of the measured current density in relation to H_2_O_2_ concentrations. It is noteworthy that the current density drastically increased as the H_2_O_2_ concentrations increased up to 200 μM, after which a modest increase in the current density occurred as the concentration increased from 200 μM up to 700 μM. Consequently, two distinct sensing behaviour regions were observed: the first ranged up to a H_2_O_2_ concentration of 200 μM, and the second covered the concentration range from 200 μM to 700 μM. The sensitivity of the prepared AZO/ZnO NR electrode was calculated from the calibration curve, using the slope found in Figure 4. In addition, the results of the lower detection limits (LODs) were based on the ratio of the standard deviation (SD) of the response and the slope of the calibration curve at a signal-to-noise ratio (SNR) of 3 [12]:

(2) $$LOD=\frac{3SD}{S}$$

where *SD* is the standard deviation of the blank and *S* is the sensitivity (the slope of the line). For the low range of H_2_O_2_ concentration (from 10 μM to 200 μM), the sensitivity was approximately equal to 1.1 μA·μM^−1^·cm^−2^ (correlation coefficient (R^2^) = 0.995), with LOD approximately equal to 42 μM. For the higher range of H_2_O_2_ concentration (from 200 μM to 700 μM), however, the sensitivity was approximately equal to 295 nA·μM^−1^·cm^−2^ (R^2^ = 0.998), with LOD close to 143.5 μM.

The inflection in the response at low and high H_2_O_2_ concentrations is due to the adsorption process on the surface of the ZnO NRs. The available surface sites for adsorption at the surface of the prepared ZnO NRs will decrease because of the increase in the amount of available H_2_O_2_. Consequently, the number of conduction centres will decrease as the chemisorption process becomes dominant, which will result in the decrease of the charges responsible for the electrical conduction because the physisorption rate will be slower [12,33]. In view of the results obtained in this work, we believe that H_2_O_2_ undergoes an oxidation process more often than a reduction process, as has been noticed by several research groups that prepared non-enzymatic H_2_O_2_ biosensors based on metal-oxide semiconductors [6,8,34,35,36]. This is probably due to the different measurement configurations.

#### 3.2.2. Amperometric Response

Figure 4 shows the typical amperometric response (current vs. time) of the AZO/ZnO NR electrode with successive increments of H_2_O_2_ added into the stirred H_2_O_2_/DI solution to achieve concentrations of 10 μM to 760 μM. The electrodes were supplied with a fixed bias voltage of 5 V. The stable and measurable currents for each step are noteworthy. The AZO/ZnO NR electrode responded rapidly as the concentration of H_2_O_2_ was increased, with a response time of less than 7 s. The substantial increase in the measured current reveals a good sensing response toward the addition of H_2_O_2_, which demonstrates the high electrocatalytic activity behaviour of the prepared ZnO NRs.

Metal oxide semiconductors are widely used as non-enzymatic sensors for H_2_O_2_. However, past measurements were based mostly on cyclic voltammetry with a three-electrode electrochemical cell. In the present study, the ZnO NR-based H_2_O_2_ sensor is investigated by simple and reliable I-V characteristics measurement. Although the results of the sensing behaviour found in this study may differ from those published in previous studies using cyclic voltammetry measurements, they are still comparable.

A number of other types of non-enzymatic H_2_O_2_ sensors have been showcased in previous studies. CuO_2_ nanostructures prepared with reduced graphene oxide (nanocomposites) have been used for non-enzymatic H_2_O_2_ sensor applications [11] and results revealed a wide measurement range for H_2_O_2_, from 30 μM to 12.8 mM with a sensitivity of 19.5 μA/mM. A TiO_2_/MWNCT electrode has also been deployed for enzyme-less sensing of H_2_O_2_ [37] and was able to detect H_2_O_2_ concentrations up to 15 mM with a sensitivity of 13.4 μA/mM and detection limit of 0.4 μM. Another H_2_O_2_ sensor employed Co_3_O_4_ nanoparticles and non-enzymatic glucose [38] and exhibited a detection limit of 0.81 μM with a high sensitivity of 107.4 μA·mM^−1^·cm^−2^. The response range, however, was up to 200 mM, which is lower than what was estimated in the present study. Despite the fact that the sensor platform was simple in the present work, the results obtained by this study are within the range of those presented by other published studies.

## 4. Conclusions

ZnO NRs for non-enzymatic sensor applications were successfully synthesised on an AZO conductive substrate via the hydrothermal process. Structure and the sensing properties of the prepared ZnO NRs were investigated using XRD, and I-V characterisations. The prepared sensor was capable of generating different responses with different H_2_O_2_ concentrations. This shows the excellent electrocatalytic activity of the prepared AZO/ZnO NR electrode in relation to H_2_O_2_, making ZnO NR a promising candidate for use in non-enzymatic sensor applications.
